# Breast cancer histology and receptor status characterization in Asian Indian and Pakistani women in the U.S. - a SEER analysis

**DOI:** 10.1186/1471-2407-10-191

**Published:** 2010-05-11

**Authors:** Madhuri Kakarala, Laura Rozek, Michele Cote, Samadhi Liyanage, Dean E Brenner

**Affiliations:** 1 Division of Hematology/Oncolog, Department of Internal Medicine, University of Michigan, 2150 Cancer Center, 1500 E Medical Center Drive, Ann Arbor, MI 48109-5390, USA; 2 V.A. Medical Center, 2150 Cancer Center, 1500 E. Medical Center Dr., Ann Arbor, MI 48109-5390, USA; 3 School of Public Health, Environmental Health Sciences, 6630 SPH 1, Ann Arbor, MI 48109-2029, USA; 4 Department of Pharmacology, University of Michigan, 2150 Cancer Center, 1500 E Medical Center Drive, Ann Arbor, MI 48109-5390, USA

## Abstract

**Background:**

Recent reports suggest increase in estrogen receptor (ER), progesterone receptor (PR) negative breast cancer yet little is known about histology or receptor status of breast cancer in Indian/Pakistani women.in the U.S.

**Methods:**

We examined the United States National Cancer Institute's Surveillance Epidemiology and End Results (SEER) Cancer program to assess: a) frequency of breast cancer by age, b) histologic subtypes, c) receptor status of breast cancer and, d) survival in Indians/Pakistanis compared to Caucasians. There were 360,933 breast cancer cases diagnosed 1988-2006. Chi-Square analyses and Cox proportional hazards models, to estimate relative risks for breast cancer mortality after adjusting for confounders, were performed using Statistical Analysis Software 9.2.

**Results:**

Among Asian Indian/Pakistani breast cancer patients, 16.2% were < 40 yrs. old compared to 6.23% in Caucasians (p < 0.0001). Asian Indian women had more invasive ductal carcinoma (69.1 vs. 65.7%, p < 0.0001), inflammatory cancer (1.4% vs. 0.8, p < 0.0001) and less invasive lobular carcinoma (4.2% vs. 8.1%, p < 0.0001) than Caucasians. Asian Indian/Pakistani women had more ER/PR negative breast cancer (30.6% vs. 21.8%, p = 0.0095) than Caucasians. Adjusting for stage at diagnosis, age, tumor grade, nodal status, and histology, Asian Indian/Pakistani women's survival was similar to Caucasians, while African Americans' was worse.

**Conclusions:**

Asian Indian/Pakistani women have higher frequency of breast cancer (particularly in age < 40), ER/PR negative invasive ductal and inflammatory cancer than Caucasians.

## Background

Breast cancer is the most frequently diagnosed cancer in females in the United States, affecting 1 in 8 women [1]. Worldwide, the incidence of breast cancer varies from 3.9/100,000 in Mozambique to as high as 101.1/100,000 in the U.S [2-5]. Geographic variation in breast cancer incidence can be attributed to racial and genetic differences, cultural differences, as well as environmental exposures that vary throughout the world [5,6]. Recent profiling work demonstrates that breast cancer is not one homogenous disease but consists of at least 5 distinct molecular subtypes with different treatment options and prognoses [7-12].

Overall incidence of breast cancer is declining in the United States in the last decade [6,13]. However, the incidence of the biologically aggressive estrogen receptor (ER) negative, progesterone receptor (PR) negative breast cancer in women younger than 40 has been increasing in African Americans in the U.S., Nigerian, Chinese, Vietnamese, and Taiwanese populations [14-16]. Recent reports from India and Pakistan suggest an important increase in the incidence of breast cancer and specifically ER, PR negative breast cancer among these populations [16-19]. ER, PR negative breast cancer, of which 50% is also Her2Neu receptor negative (triple negative), is biologically aggressive, resistant to conventional cytotoxic chemotherapy treatment, and is associated with reduced survival compared to other subtypes of breast cancer [20-23].

Cancer incidence studies in Asian Indians and Pakistanis in India and Pakistan as well as emigrants to various countries including Canada, United States, Singapore, United Kingdom have documented a rise in breast cancer in premenopausal Indian and Pakistani women (younger than 40) compared to local Caucasian women [24-33]. Yet very little is known about the specific histologic subtypes or receptor status of breast cancer in women of Indian/Pakistani origin in the U.S. [34-38]. Understanding frequency of occurrence of specific breast cancer subtypes and associated risk factors in Indians/Pakistanis may elucidate breast cancer prevention, screening and treatment strategies tailored to the unique risk of this ethnic group.

We, therefore, explored whether analysis of United States National Cancer Institute's Surveillance Epidemiology and End Results (SEER) Program would: a) indicate a disproportionately high frequency of occurrence of breast cancer in Asian Indian/Pakistani women younger than 40 yrs (premenopausal age) compared to Caucasian females, b) provide data on specific histologic subtypes of breast cancer (eg. invasive ductal, inflammatory or lobular carcinoma), and c) molecular subtypes of breast cancer by receptor status in Asian Indian and Pakistani women in the United States and, 4) the impact of these subtypes on breast cancer specific survival. In this exploratory analysis, we examined demographic characteristics such as age and marital status and biological variables such as histology, estrogen and progesterone receptor status and in situ versus invasive disease as predictive variables for disease outcome and survival.

## Methods

Subjects were 360,933 females, identified as Caucasian, African American, Hispanic or Indian/Pakistani, diagnosed with breast cancer between 1988 and 2006. Information regarding these subjects was obtained from the population-based SEER database in a case listing session. The SEER program collects information about all incident cancer cases including patient demographics, tumor site, stage at diagnosis, first course of treatment and annual follow up for vital status (SEER website: http://seer.cancer.gov/about/). Currently, 18 population-based registries cover 26% of the United States population. Overall, the combined registries are comparable to the rest of the United States population with regard to poverty and education levels, but are slightly more urban and contain a higher proportion of foreign-born individuals. For the purposes of this analysis, data from 6 SEER sites with high proportion of Indian/Pakistani women were used. These 6 sites are: Atlanta--Georgia, Connecticut, California (Los Angeles, San Francisco-Oakland, San Jose-Monterey, and greater California), Metropolitan Detroit--Michigan, New Jersey, and Seattle-Puget Sound--Washington. In the SEER database, histological type of tumor is coded using International Classification of Diseases for Oncology, Third Edition (ICD-O) codes. The corresponding ICD-O morphology codes for breast cancers (Site and Morphology code = 'Breast') were selected: 8530 (inflammatory), 8522-8524 (mixed), 8100, 8500, 8501, 8503, 8521 and 8523 (ductal), and 8520 (lobular). Two behavior codes were also selected: 2 (in situ) and 3 (invasive). For the purposes of this analysis, a categorical variable was created, with categories for each histological type and behavior. Variables of interest included age at diagnosis, marital status, registry site, AJCC stage, tumor markers (ER and PR), vital status and survival time (months). Population denominators were not available for the Indian/Pakistani population in SEER or in United States Census data, so these results represent frequencies, not rates. As these data are de-identified, human investigation approval was not necessary for this project, but all investigators have signed limited-use data agreements to access and analyze SEER data.

### Statistical Methods

Bivariate analyses (PROC FREQ) and multinomial analyses (PROC CATMOD) were implemented using Statistical Analysis Software (SAS) version 9.2. The p-values presented represent comparisons between Caucasian and Indian/Pakistani breast cancer patients. Bivariate analyses compared categorical age at diagnosis, marital status and SEER program site by race. Multinomial analyses compared clinical characteristics (histology, AJCC SEER modified stage, grade, ER/PR status, tumor size and nodal status) by race adjusting for age and SEER program site. For age, we assigned three age categories: age < 40 yrs corresponding to premenopausal, age 40-50 yrs. for perimenopausal and age > 50 for post menopausal age groups. These are empirical groupings and not based upon hormone testing to establish menopausal status. Tumor size was categorized as < 1 cm, 1 - 4 cm and > 4 cm.

### Hormone Receptor Status Analysis

Estrogen receptor was available for 50.5% and progesterone receptor status was available for 49.8% of all breast cancer cases, including in situ ductal or lobular carcinoma (n = 204), invasive ductal (n = 817), invasive lobular (n = 50) and inflammatory breast cancer (n = 16) in Asian Indian/Pakistani females (n = 1087). The proportion of missing data did not differ between Caucasian, Indian/Pakistani, Hispanic and African-American breast cancer patients.

### Survival

Data from all 6 SEER registries were used for survival analyses. We chose to analyze from 1988 forward because prior to 1988 Asian Indians and Pakistanis were identified only as "Other Asians". Cox proportional hazards models were used to estimate relative risks (RRs) and 95% Confidence Intervals for mortality from breast cancer after adjusting for relevant confounders including AJCC SEER modified stage, histology, behavior, age at diagnosis and SEER program site. A minimum survival time of 2 months was used in selecting cases in order to exclude those diagnosed only at autopsy or those who may have died before pursuing treatment. Non Hispanic Caucasians served as the reference group for relative risk comparisons. All tests were two tailed and p values < 0.05 were considered statistically significant.

## Results

### Frequency of Breast Cancer in Asian Indians/Pakistanis

A total of 1350 cases of breast cancer cases among Asian Indian/Pakistani women were found in the SEER database from 6 SEER sites between 1988-2006. Among Asian Indian/Pakistani breast cancer patients, 16.2% were women younger than age 40 or premenopausal women compared to 6.23% in Caucasian women (p < 0.0001) and 10.8% of African American and 11% of Hispanic women as shown in Table 1. This higher percentage of women under age 40 with breast cancer diagnoses in Asian Indians/Pakistanis was also found in the perimenopausal 40-50 yr olds as well with 29.9% of Asian Indians/Pakistanis compared to 18.9% of Caucasian women (p < 0.0001). African American and Hispanic women between ages 40-50 had intermediate frequency with 23.1% and 24.6% respectively. A correspondingly lower percentage of breast cancer was found in postmenopausal (age > 50 yrs.) Asian Indian/Pakistani women compared to Caucasians (53.9% vs. 75.5%, p < 0.0001).

**Table 1:** Age at diagnosis, marital status and survival of women with breast cancer, 6 SEER program sites, 1988-2006, by ethnicity

Variable	Caucasian n = 300,494	Indian/Pakistani n = 1,350	African-American n = 40,647	Hispanic n = 18,442	P value*
**Age category**					< 0.0001
Premenopausal (age < 40)	18001 (6.0)	219 (16.2)	4405 (10.8)	2026 (11.0)	
Perimenopausal (age 40-50)	55597 (18.5)	403 (29.9)	9401 (23.1)	4536 (24.6)	
Postmenopausal (age > 50)	226896 (75.5)	728 (53.9)	26841 (66.0)	11880 (64.4)	

**Marital Status**					< 0.0001
Single	33145 (11.0)	92 (6.8)	9528 (23.4)	3080 (16.7)	
Married	166927 (55.6)	1005 (74.4)	14537 (35.8)	9814 (53.2)	
Other	100422 (33.4)	253 (18.7)	16582 (40.8)	5548 (30.1)	

**SEER Program Site**					< 0.0001
San Franciso-Oakland	44281 (14.7)	312 (23.1)	5129 (12.6)	3392 (18.4)	
Connecticut	53693 (17.9)	136 (10.1)	3293 (8.1)	1743 (9.5)	
Metropolitan Detroit	49462 (16.5)	141 (10.4)	12416 (30.6)	466 (2.5)	
Seattle (Puget Sound)	54259 (18.1)	84 (6.2)	1378 (3.4)	476 (2.6)	
Metropolitan Atlanta	23223 (7.7)	131 (9.7)	8547 (21.0)	467 (2.5)	
San Jose-Monterey	18291 (6.1)	238 (17.6)	412 (1.0)	1679 (9.1)	
Los Angeles	57285 (19.1)	308 (22.8)	9472 (23.3)	10219 (55.4)	

Mean Survival in Months (SD)	67.0 (53.7)	48.0 (46.2)	53.0 (52.7)	56.0 (49.5)	< 0.0001**

*p < 0.0001 for difference between Asian Indian/Pakistani women and Caucasian women.

Survival analysis here is univariate by ethnicity. Multivariate analysis including histologic, molecular and other disease and patient variables is presented in the results text.

**Wilcoxon rank-sum test between Indian/Pakistani and Caucasian median survival

### Breast Cancer Stage at Diagnosis in Asian Indians/Pakistanis

Percentage of women diagnosed with stage IV or metastatic disease was similar in Asian Indians/Pakistanis compared to Caucasians (4.9 vs. 4.5%). African American and Hispanic women had significantly higher percentage of women presenting with stage III (11.9% and 9.6% versus 7.0%, p < 0.01) compared to Caucasian women. African American women also had the highest percent of stage IV disease (8.1% compared to 4.5% in Caucasians, p < 0.01).

### Breast Cancer Histology and Receptor Status in Asian Indians/Pakistanis

The histologic subtypes of in situ lobular or mixed in situ cancer differed significantly between Asian Indian/Pakistani compared to Caucasian women, while in situ ductal cancer did not differ significantly as shown in Table 2 (p = 0.02), however, given the large number of subjects the significance is not likely meaningful. Asian Indian women had a slightly higher frequency of invasive ductal carcinoma (69.1 vs. 65.6%, p < 0.0001) and lower invasive lobular carcinoma (4.2% vs. 8.2%, p < 0.0001) compared to Caucasian women. Mixed histology tumors were of similar frequency of occurrence in both ethnic groups. Inflammatory breast cancer, which is a particularly aggressive subtype, was slightly higher in Asian Indian/Pakistanis, African Americans and Hispanics compared to Caucasians (1.4%, 1.5%, 1.6% respectively vs. 0.8%, p < 0.0001). More Asian Indian/Pakistani women were diagnosed at AJCC Seer Modified Stage II (47.5% vs. 37.5%, p < 0.001), III (11.3% vs. 7.0%, p < 0.001) and a smaller percentage in stage I (36.3% vs. 51.0%, p < 0.001) compared to Caucasian women as shown in Table 3. There was higher percentage of estrogen and progesterone receptor negative breast cancer (30.6% vs. 21.8%, p = 0.0095) breast cancer in Asian Indian/Pakistani women compared to Caucasians (Table 2). Hispanics were similar to Asian Indians/Pakistanis in receptor negativity (29.7 vs. 30.6%) but African Americans had the highest percentage of ER/PR negative disease 41% compared to 21.8% in Caucasians (p < 0.0095). We explored marital status of breast cancer patients and found that a significantly greater percentage of Asian Indian/Pakistani than Caucasian breast cancer patients are married (74.4% vs. 55.4%, p < 0.0001).

**Table 2:** Histology and receptor status of breast cancer in women in the 6 SEER program sites diagnosed between 1988-2006 by ethnicity

Variable	Caucasian	Indian/Pakistani	African-American	Hispanic	P value*
**Histology**					0.02*
In situ---Ductal	31998 (12.1)	142 (12.0)	4409 (12.7)	1907 (11.8)	
In situ---Lobular	6641 (2.5)	21 (1.8)*	648 (1.9)	330 (2.0)	
In situ--Mixed	6243 (2.4)	41 (3.5)*	984 (2.8)	420 (2.6)	

**Histology**					< 0.0001*
Invasive--Ductal	173174 (65.6)	817 (69.1)*	24254 (69.6)	10858 (67.3)	
Invasive---Lobular	21725 (8.2)	50 (4.2)*	1752 (5.0)	1023 (6.3)	
Invasive--Mixed	21853 (8.3)	96 (8.1)	2253 (6.5)	1322 (8.2)	
Inflammatory carcinoma	2218 (0.8)	16 (1.4)*	537 (1.5)	265 (1.6)	

**Estrogen Receptor (ER)**					0.0688*
Positive	130267(79.3)	463 (71.9)*	12902 (62.6)	6984 (72.3)	
Negative	34040 (20.8)	181 (28.1)*	7724 (37.5)	2673 (27.7)	

**Progesterone Receptor (PR)**					0.0260*
Positive	108451(68.2)	391 (62.2)*	10548 (53.1)	5758 (62.5)	
Negative	50533 (31.8)	238 (37.8)*	9315 (46.9)	3449 (37.5)	

**ER/PR**					0.0095*
Positive/Positive	103767 (78.2)	377 (69.4)*	9702 (58.9)	5408 (70.3)	
Negative/Negative	28994 (21.8)	166 (30.6)*	6772 (41.1)	2285 (29.7)	

* p value is for comparison between Asian Indian/Pakistani and Caucasian women adjusted for age at diagnosis and SEER program

**Table 3:** Stage at diagnosis, tumor grade, primary tumor size and nodal status by ethnicity in 6 SEER program sites between 1988-2006.*

Variable	Caucasian	Indian/Pakistani	African-American	Hispanic	P value*
**AJCC stage**					< 0.001
Stage I	118856 (51.0)	381 (36.3)*	11187 (35.9)	6069 (41.9)	
Stage II	87267 (37.5)	499 (47.5)*	13735 (44.1)	6322 (43.7)	
Stage III	16236 (7.0)	119 (11.3)*	3698 (11.9)	1394 (9.6)	
Stage IV	10619 (4.5)	51 (4.9)	2512 (8.1)	694 (4.8)	

Tumor Grade					< 0.0001
1	42232 (19.0)	150 (13.5)*	3751 (12.5)	2191 (15.0)	
2	93718 (42.2)	452 (40.8)	10376 (34.6)	5751 (39.5)	
3	76391 (34.4)	465 (41.9)*	14561 (48.6)	5856 (40.2)	
4	9607 (4.3)	42 (3.8)	1254 (4.2)	758 (5.2)	

Primary Tumor Size					0.4432
< 1 cm	8364 (25.4)	51 (21.4)	938 (18.2)	564 (20.5)	
1 -- 4 cm	20748 (62.9)	151 (63.5)	3140 (60.9)	1758 (64.0)	
> 4 cm	3887 (11.7)	36 (15.1)	1079 (20.9)	425 (15.5)	

**# of nodes positive**					0.4668
0	144518 (68.6)	597 (59.5)	16291 (60.4)	8459 (63.1)	
1-3	42546 (20.2)	236 (23.5)	6474 (24.0)	2979 (22.2)	
4-9	15256 (7.2)	111 (11.1)	2708 (10.1)	1242 (9.3)	
> 9	8325 (4.0)	60 (5.9)	1481 (5.5)	718 (5.4)	

* p value is for comparison between Asian Indian/Pakistani and Caucasian women adjusted for age at diagnosis and SEER program

### Breast Cancer Specific Survival in Asian Indians/Pakistanis

In order to better understand the correlates of survival in racial subgroups, we further examined this survival outcome in multivariate regression analysis using age at diagnosis, AJCC SEER Modified Stage, and histologic subtype as predictors for survival (Table 4). Survival time was censored at five years. Estrogen/progesterone receptor status and nodal status were not used as their data were sparse in the younger age groups in minority populations. Women with inflammatory breast cancer were found to have a hazard ratio (HR) of 3.47 (CI: 2.48 - 4.86), increasing stage HR of 2.80 (CI: 2.63-2.98), African Americans HR of 1.59 (CI: 1.37-1.85), and older age group at diagnosis a HR of 2.0 (CI: 1.75-2.29) of not surviving to 5 years post diagnosis compared to Caucasian women. Once stage at diagnosis, age, and histologic profile were controlled for, the survival outcome of Asian Indian/Pakistani women did not differ significantly from Caucasians (HR = 0.45, C.I. = 0.11 - 1.79) however, African American women had significantly worse survival than Caucasians.

**Table 4:** Disease-specific five- year survival using Cox-proportional hazards adjusted for SEER site

Predictor	Univariate HR (95% C.I.)	Multivariate model
Stage	3.08 (2.905 -- 3.26)	2.80 (2.63 -- 2.98)

Inflammatory	5.41 (5.12 -- 5.71)	3.47 (2.48 -- 4.86)

Mixed	0.73 (0.70 -- 0.77)	0.95 (0.70 -- 1.28)

Ductal	1.03 (1.00 -- 1.06)	1.43 (1.13 -- 1.80)

Lobular	1.00 (reference)	1.00 (reference)

African-American	1.66 (1.62 -- 1.69)	1.59 (1.37 -- 1.85)

Hispanic	0.95 (0.91 -- 0.99)	0.72 (0.52 -- 0.98)

Indian/Pakistani	0.82 (0.70 -- 0.96)	0.45 (0.11 -- 1.79)

Caucasian	1.00 (reference)	1.00 (reference)

Age group	1.39 (1.37 -- 1.41)	2.00 (1.75 -- 2.29)

## Discussion

Asian Indians/Pakistanis are one of the fastest growing ethnic groups in the United States and have a higher frequency of breast cancer than Caucasians [26,33]. Our analysis of breast cancer diagnoses using the SEER database for cases diagnosed from 1988-2006 finds a disproportionately high occurrence rate of breast cancer in Asian Indian/Pakistani women younger than 40 yrs. compared to Caucasians. It is possible the overall age distribution of Asian Indian/Pakistani women is younger compared to the other racial/ethnic groups in the United States, resulting in a disproportionate breakdown by age. This could be due to immigration patterns, as the Indian population reflects the rapid increases in young immigrants in the latter half of the 20^th ^century. Unfortunately, immigration data were not available in SEER, but our results suggest the need for future studies to address this question. However, these findings are consistent with similar high rates observed among south Asians or Indians in Singapore, United Kingdom, Malaysia, Canada and in Indians in India [25,26,33,34,39-41]. We were unable to examine trends over time using the SEER program due to the relatively small number of cases diagnosed every year for Asian Indians and Pakistanis, however at least one study shows a rise in breast cancer incidence rates in Asian Indians over time [14].

Over the past decade, the biology of breast cancer has been redefined into groups of distinct biological subtypes. Each subtype presents with specific clinical, pathological and molecular phenotypes associated with diverse natural histories, therapeutic implications and prognoses [8,42-47]. Previously published studies of breast cancer in Asian Indians did not examine the frequency of specific histologic subtypes of breast cancer: invasive ductal, lobular and inflammatory carcinoma of the breast. Each of these subtypes has a different biologic behavior and prognosis [11,48,49]. Inflammatory breast cancer, for example, is the most aggressive histologic subtype with worse hazard ratio for 5 year survival in our own multivariate analysis [50-52] and occurs more frequently among Asian Indians compared to Caucasians in our analysis. Lobular invasive carcinoma may present with bilateral disease and recur many years from primary disease presentation. The most common histology in both Caucasians and Asian Indians/Pakistanis, invasive ductal carcinoma is usually unilateral and risk of recurrence decreases as time elapses from primary diagnosis [49,53,54]. Differences in behavior of distinct breast cancer histologies determine whether a patient receives breast conservation or aggressive surgical treatment, sentinel node or complete axillary dissection. Systemic treatment such as duration of hormone therapy, targeted therapy for Her2Neu positive disease and followup patterns also differ for different breast cancer subtypes [55]. Our SEER analysis shows that invasive ductal carcinoma is more frequently diagnosed in Asian Indian/Pakistani women compared to Caucasian women while diagnosis of lobular carcinoma is correspondingly lower.

A higher proportion of breast cancer in Asian Indian/Pakistani women compared to Caucasians (30.6% vs. 21.8%, p < 0.0095) in the SEER database was ER and PR negative. It must be noted that individuals for whom receptor status was missing were excluded from that analysis and missing data was comparable in both groups. Reports in Asian Indians in India and the United Kingdom also document high rates of ER, PR negative breast cancer [34,36]. Interestingly, when receptor status was examined by age, we found that this difference was driven by increased percentage of ER/PR negative disease in 40-50 yr olds and those older than 50, rather than by younger aged women.

We were unable to examine Her2 Neu receptor status as this variable is only recently being collected by the SEER program. However, pathologic assessments of tumors over time have found the particularly aggressive, and least treatable, triple negative (ER, PR and Her2Neu negative) or basal breast cancer molecular subtype to be common in Asian Indians [38,56]. This subtype of breast cancer is more common among BRCA1 gene mutation carriers, however, known BRCA1 and BRCA2 mutations do not seem to explain the triple negative cancer in Indian women [57-59]. Stage at diagnosis is critical in breast cancer as early stage cancer is curable while more advanced stages are not [28,60]. Asian Indian/Pakistani women were diagnosed at significantly more advanced stages of breast cancer than Caucasian women suggesting a need to raise awareness of screening recommendations in this ethnic group.

## Conclusion

In conclusion, Asian Indian and Pakistani women in the US had more ER and PR negative tumors than their Caucasian counterparts. Invasive ductal carcinoma is the most common histology. Inflammatory cancer is also more common in Asian Indians/Pakistanis than Caucasians. Given the limited treatment options for receptor negative breast cancer and poor prognosis, it is important to encourage screening and early diagnosis measures such as annual clinical breast exams for these women.

## Competing interests

The authors declare that they have no competing interests.

## Authors' contributions

MK, LR, MC SL and DB conceptualized this study, MK and LR conducted the analysis, MC extracted the SEER data and interpreted each variable, SL conducted background literature review and assisted with analysis, all authors wrote this manuscript and approved the final version.

## Pre-publication history

The pre-publication history for this paper can be accessed here:

http://www.biomedcentral.com/1471-2407/10/191/prepub
